# Eliminating malaria and preventing its reintroduction: the Mauritius case study

**DOI:** 10.1186/1475-2875-11-S1-O12

**Published:** 2012-10-15

**Authors:** Shahina Aboobakar, Allison Tatarskv, Justin M Cohen, Ambicadutt Bheecarry, Premnath Boolaky, Neerunjun Gopee, Devanand Moonasar, Allison A Phillips, James G Kahn, Bruno Moonen, David L Smith, Oliver Sabot

**Affiliations:** 1 Ministry of Health and Quality of Life, Port Louis, Mauritius; 2 Clinton Health Access Initiative, Boston, MA, USA; 3 The Global Health Group, University of California, San Francisco, San Francisco, CA, USA; 4 National Department of Health, Pretoria, South Africa; 5 Department of Epidemiology and Biostatistics, University of California, San Francisco, San Francisco, CA, USA; 6 Emerging Pathogens Institute, FL 32610, USA; 7 Department of Biology, University of Florida, Gainesville, FL, USA

## 

This abstract is submitted as part of the panel session on case studies for elimination by the WHO Global Malaria Programme and the UCSF Global Health Group.

## Background

Sustaining elimination of malaria in areas with high receptivity and vulnerability will require effective strategies to prevent reestablishment of local transmission, yet there is a dearth of evidence about what such approaches should involve. Mauritius offers a uniquely informative history, with elimination of local transmission in 1969, reemergence in 1975, and second elimination in 1998.

## Materials and methods

To provide evidence for future elimination programs, Mauritius’s elimination and prevention of reintroduction (POR) programs were analyzed through a comprehensive review of literature and government documents, supplemented by program observation and interviews with policy makers and program personnel. The impact of the country’s most costly intervention, a passenger screening program, was assessed quantitatively using simulation modeling.

## Results

Following the introduction of malaria in Mauritius in the mid-1800s, *P. vivax* and *P. falciparum* malaria were hyperendemic until the government launched an aggressive campaign to interrupt transmission and eliminate the parasite through indoor residual spraying (IRS) in 1948. Between 1948 and 1963, incidence rates declined from 105 cases per 1,000 population at risk to 0.04 at an estimated cost of $5.75 per capita per year (pcpy) between 1948 and 1949 and $2.99 pcpy between 1960 and 1961. *Anopheles funestus was* eliminated during this time, leaving *An. gambiae* as the main vector.

Local *P. vivax* transmission was reestablished in 1975 after large cyclones created new breeding sites and parasitaemic workers from endemic countries arrived to rebuild the damaged infrastructure. Lax interventions (e.g., surveillance and vector control) during the first POR program may have also contributed to this resurgence, as well as increased importation risk.

Mauritius launched a second elimination campaign from 1982 to 1988 through implementation of a combination of focal interventions, widespread larviciding, and an extensive case response system at a cost of $4.43 pcpy. The country currently spends $2.06 pcpy on its POR program that includes robust surveillance, routine vector control (larviciding island-wide and IRS at the ports of entry), free chemoprophylaxis to travelers, and prompt and effective diagnosis, treatment, and response. Thirty-five percent of POR costs are for a passenger screening program through which passengers arriving from malaria endemic countries, report having been in an endemic country in the last six months, or who are febrile upon or soon after arrival are tested at the ports of entry or are contacted by surveillance officers at their residence. Between 2005 and 2008, an average of 42,612 blood smears collected through passenger screening were examined for malaria parasites detecting an average of 10 positive cases each year. Modeling suggests that the estimated 14% of imported malaria infections identified by this program reduces the annual risk of local transmission by approximately 2%.

## Conclusion

The Mauritius experience demonstrates that it is possible to eliminate malaria and prevent its reintroduction in a country with relatively high receptivity and moderate vulnerability but that continuous vigilance and some control to reduce and maintain low vector density is critical. Strong leadership and substantial predictable funding are critical to consistently prevent resurgence in Mauritius and must be sustained.

